# Granulocyte colony stimulating factor treatment in non-alcoholic fatty liver disease: beyond marrow cell mobilization

**DOI:** 10.18632/oncotarget.18967

**Published:** 2017-07-04

**Authors:** Ho Hyun Nam, Dae Won Jun, Kiseok Jang, Waqar Khalid Saeed, Jai Sun Lee, Hyeon Tae Kang, Yeon Ji Chae

**Affiliations:** ^1^ Department of Translational Medicine, Hanyang University Graduate School of Biomedical Science and Engineering, Seoul, South Korea; ^2^ Department Internal Medicine, Hanyang University School of Medicine, Seoul, South Korea; ^3^ Department of Pathology, Hanyang University School of Medicine, Seoul, South Korea

**Keywords:** non-alcoholic fatty liver, granulocyte colony stimulating factor, apoptosis

## Abstract

Protective effects of granulocyte colony stimulating factor (G-CSF) in acute liver injury via marrow cell mobilization have been reported in several studies. But exact mode of action and optimal protocol of G-CSF has been still doubt in chronic disease. Here we investigated mode of action and optimization of G-CSF as a treatment for non-alcoholic fatty liver disease (NAFLD). Various doses of conventional G-CSF (30 μg/kg once weekly, once daily for 5 days, twice weekly) and long acting G-CSF (30 μg/kg once a month) were evaluated in two kinds of NAFLD animal models to optimize the G-CSF protocol. G-CSF receptor expression highest increased in NAFLD model among various liver diseases compare to control (NAFLD: 14.7 times, alcohol hepatitis: 7.1 times, cirrhosis: 2.4 times, and ischemia reperfusion: 6.8 times). G-CSF treatment reduced intrahepatic fat accumulation, and inflammation in two kinds of NAFLD animal models. G-CSF increased PI3K/Akt expression in hepatocyte as well as decreased apoptotic drive (increased Bcl-2 expression and decreased Bax expression) in animal model. Five day consecutive G-CSF treatment and once a month long acting G-CSF increased marrow derived stem cell marker in peripheral blood. But twice a week conventional G-CSF treatment did not increased CD34+ cell in peripheral blood and liver neither. Not only high dose G-CSF (once daily for 5 days) but also hepatotropic dose G-CSF (twice a week) significantly reduced hepatocyte apoptosis via PI3K and Akt pathway activation without marrow cell mobilization in NAFLD animal model.

## INTRODUCTION

The prevalence of non-alcoholic fatty liver disease (NAFLD) is 20–30% [1–3], and 10–20% of which is accompanied by steatohepatitis.

In previous several reports the granulocyte colony stimulating factor (G-CSF), a protein containing 175 amino acids, had protective effects on various disease models via mobilization of bone marrow stem cell to damaged organs [4–8]. G-CSF mobilized marrow cells to the area of myocardial infarction where the stem cells reduce the size of infarct by helping myocardial vessels regeneration and thereby lowering the mortality by 68% [5]. Similarly, the high dose G-CSF induced bone marrow stem cell mobilization and therefore, the protective effects have been observed in a number of liver diseases [4, 6–8]. For instance, five-day G-CSF treatment decreased hepatic inflammation and increased survival in D-galactosamine-induced acute hepatic injury model [8]. The bone marrow transplantation and G-CSF administration improved hepatic albumin synthesis The G-CSF enhanced marrow cell homing to damaged liver in carbon tetrachloride induced acute liver injury model [4]. G-CSF also increased hepatic regeneration by facilitating the migration of marrow derived stem cell in partial hepatectomy model [9]. G-CSF administration not only reduced Child-Turcotte-Pugh, model for end-stage liver disease score but also increased 90-day survival rate in severe alcoholic hepatitis patients [7].

Interestingly, there is no data whether G-CSF could attenuate intrahepatic inflammation and hepatocyte apoptosis in steatohepatitis, not just only the simple steatosis. Moreover, optimal dose and schedule of G-CSF was also unclear. Most of previous studies used five-day consecutive G-CSF treatment to mobilize marrow stem cell and/or endogenous oval cell in acute liver disease. As, the steatohepatitis is a chronic disease, therefore clinical treatment strategy that would warrant the beneficial effects needs to be optimized in chronic diseases.

Although a number of studies suggested the protective effects of G-CSF administration in acute liver failure model; however, the optimal treatment strategy for G-CSF administration was still unclear. G-CSF was administered 4–5 times a week in most studies, because the protective effects were assumed to be caused by homing of mobilized BMSCs in damaged liver. However, the potential mechanisms involved in hepatotropic effects of G-CSF that promote liver regeneration and repair has never been extensively studied in liver diseases. Repeated high-dose G-CSF was administration 4–5 times a week mobilizes bone marrow stem cells which might cause complications including splenic rupture, acute lung injury, vascular events and exacerbation of autoimmune or inflammatory conditions [10]. Therefore, the suitable treatment strategy in chronic diseases needs to be optimized.

Therefore, current study aimed to evaluate the protective mechanism of G-CSF beyond the marrow cell mobilization and to clinically optimized G-CSF treatment strategy in NAFLD.

## RESULTS

### G-CSFr expression in various liver disease models

**Figure 1:**
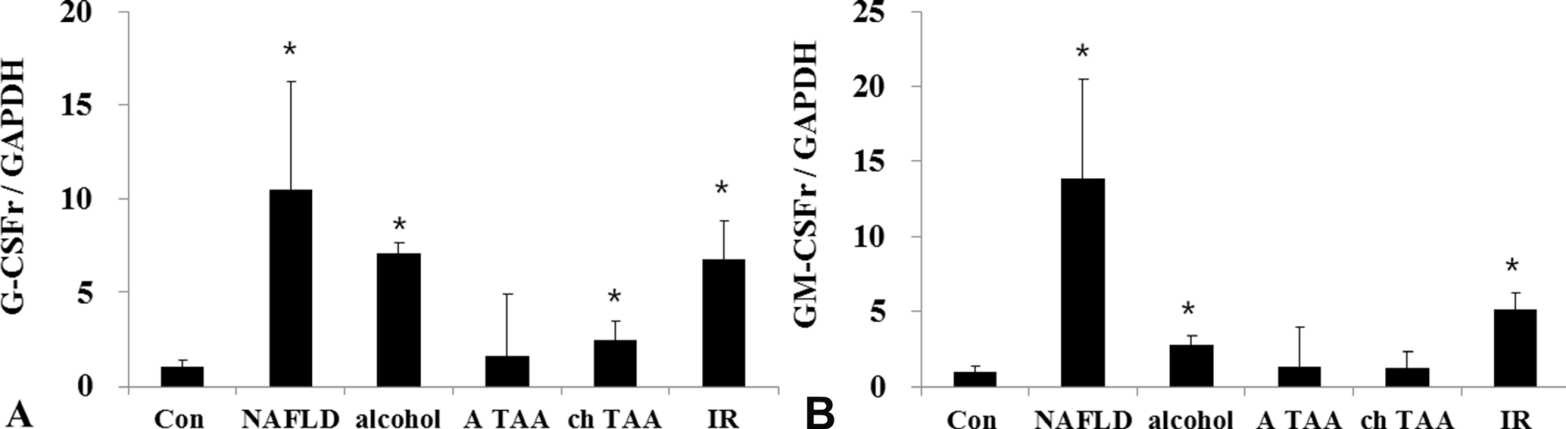
G-CSFr and GM-CSFr PCR expression in various liver disease models G-CSFr was increased in NAFLD (*n* = 10), Alcohol (*n* = 10), ch TAA (*n* = 10), and IR models (*n* = 10) (**A**). GM-CSFr was increased in NAFLD, Alcohol, IR groups (**B**). ^*^p < 0.05 by ANOVA. Abbreviations: Con, Control; NAFLD, Non-alcoholic fatty liver disease model; alcohol, Alcoholic liver disease model; A TAA, Acute thioacetamide toxic injury model; ch TAA, chronic thioacetamide induced fibrosis model; IR, hepatic ischemic and reperfusion model.

G-CSFr expression was significantly increased in nonalcoholic fatty liver mice (14.7 times, *p* < 0.001), alcoholic liver disease (7.4 times, *p* = 0.004), cirrhosis model (2.4 times, *p* = 0.05) and hepatic (IR) (6.8 times, *p* = 0.005) models as compared to the control mice (Figure 1A). GM-CSF receptor (GM-CSFr) expression was also significantly increased in NAFLD, alcoholic liver disease and hepatic IR models as compared to the control mice (Figure 1B). However, GM-CSFr expression in acute toxic injury model (acute thioacetamide treatment, A-TAA) and cirrhosis (chronic thioacetamide treatment, Ch-TAA) was insignificant (Figure 1A and 1B).

### Effects of conventional G-CSF treatment on HF induced NAFLD model

**Figure 2:**
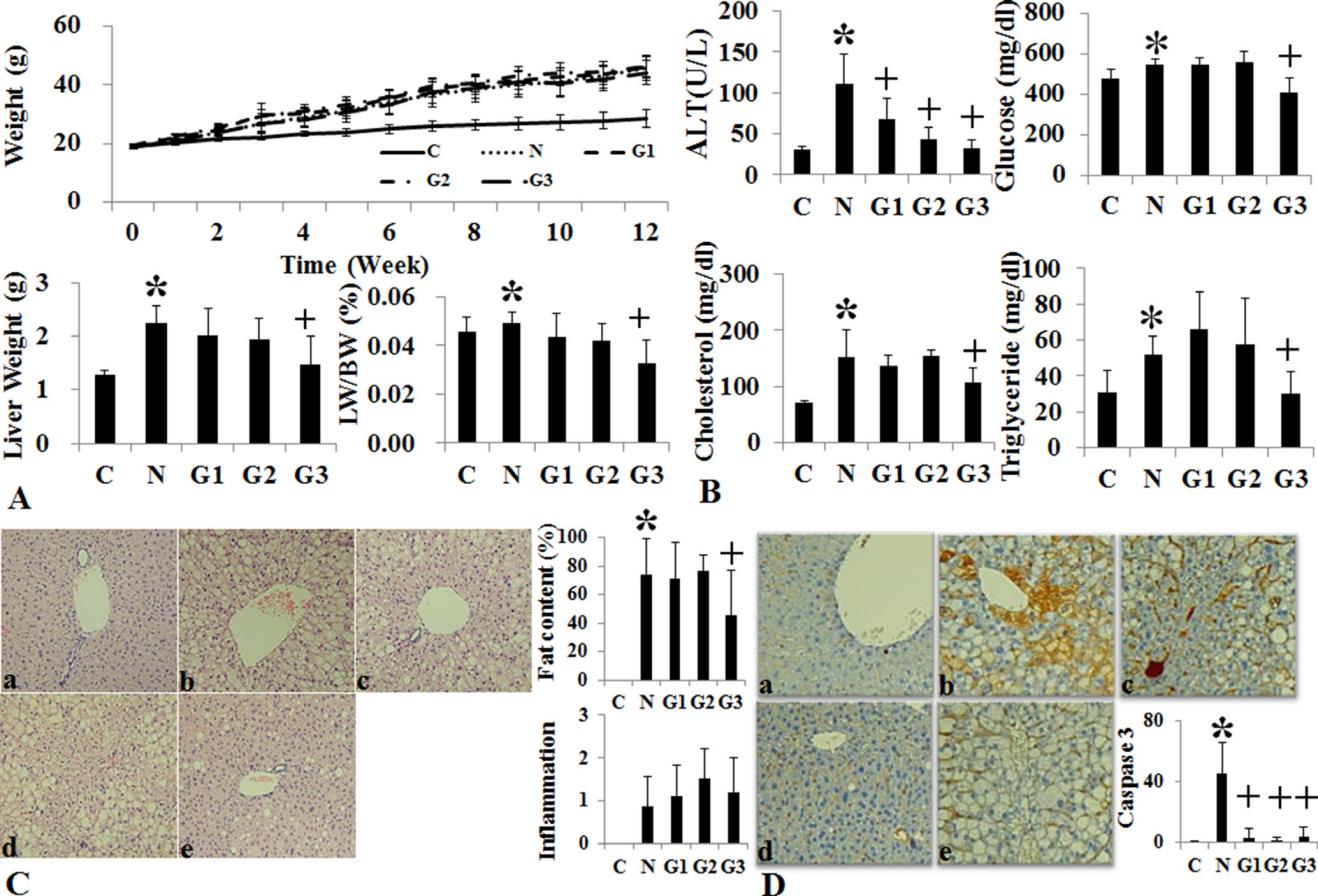
Changes of body weight, liver histology and biochemistry following conventional G-CSF treatment in high fat induced fatty liver model Changes in body weight, Liver weight and liver/body weight (LW/BW) among the control, HF and G-CSF treated groups. In G2 and G3 groups the liver weight and LW/BW ratio decreased as compared to HF diet alone group (**A**). Serum ALT in G-CSF treatment groups (G1–G3) decreased as compared to control group (**B**). Histological Changes Following G-CSF Administration. G3 (hepatotropic dose) group significantly decreased steatosis as compared to HF group (**C**). Caspase-3 immunohistochemical stain. G3 group had decreased caspase-3 staining as compared to HF group (**D**). Control group (*n* = 8), NAFLD (*n* = 8), G1 (*n* = 10), G2 (*n* = 10), G3 (*n* = 10). ^*^p < 0.05 by ANOVA with post-hoc Duncan, when it compare to control group (C), ^+^*p* < 0.05 by ANOVA with post-hoc Duncan, when it compare to NAFLD group (N). Abbreviations: C, control; N, high fat induced fatty liver disease; G1, G-CSF treatment once weekly from 8th to 12th week; G2, G-CSF treatment daily for 5 consecutive days in 9th week; G3, G-CSF treatment twice weekly from 9th to 12th week.

**Figure 3:**
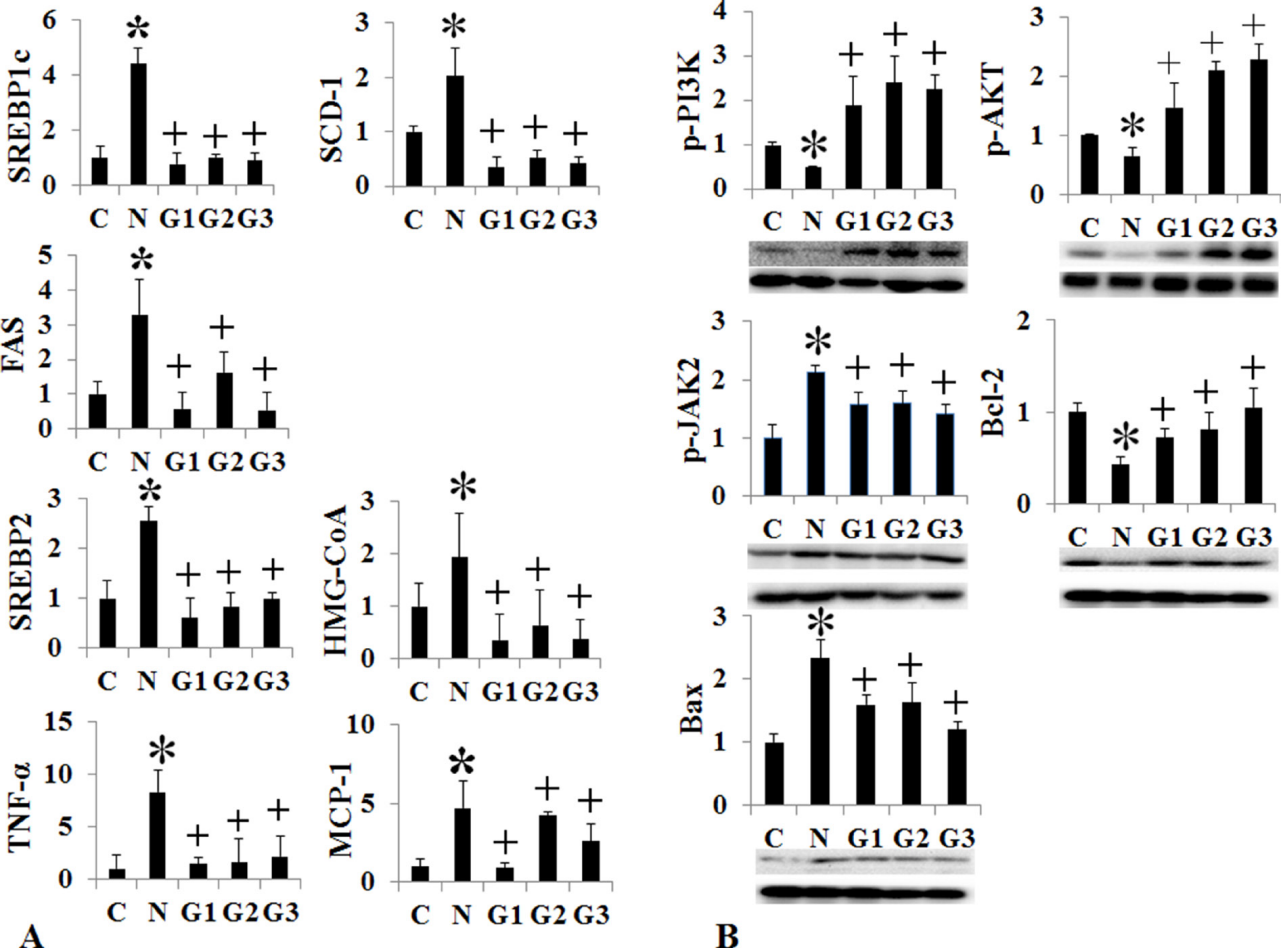
Changes of intrahepatic fat, cholesterol, inflammation, and apoptosis signal after conventional G-CSF treatment in high fat induced NAFLD model Triglyceride de novo synthesis markers (SREBP1c, SCD-1, FAS), cholesterol de novo synthesis markers (SREBP2, HMG-CoA reductase), and inflammatory markers (TNF-α, MCP-1) decreased G-CSF treatment groups (G1–G3) compared to NAFLD group (**A**). Change in cell proliferation and apoptosis marker. Bcl-2 expression increased and Bax expression decrease in G-CSF treatment groups (G1–G3) (**B**). Control group (*n* = 8), NAFLD (*n* = 8), G1 (*n* = 10), G2 (*n* = 10), G3 (*n* = 10). ^*^p < 0.05 by ANOVA with post-hoc Duncan, when it compare to control group (C), + *p* < 0.05 by ANOVA with post-hoc Duncan, when it compare to NAFLD group (N). Abbreviations: C, control; N, high fat induced fatty liver disease; G1, G-CSF treatment once weekly from 8th to 12th week; G2, G-CSF treatment daily for 5 consecutive days in 9th week; G3, G-CSF treatment twice weekly from 9th to 12th week.

G-CSF treatment to G3 group (twice a week for one month) showed significant lower liver weight and liver to body weight ratio than the HF induced NAFLD group (Supplementary Table 1) (Figure 2A). Serum ALT, glucose, cholesterol and triglyceride were all lower in G3 group (twice a week G-CSF) than the NAFLD group (Supplementary Table 2) (Figure 2B). Serum ALT was also significantly reduced in G1 and G2 groups as compared to NAFLD. The intrahepatic fat decreased in G3 group than the HF induced NAFLD group (74.3 ± 25.1% vs 45.5 ± 31.6% *p* = 0.049), while there was no significant reduction in G1 (once a week) (71.5 ± 24.9%) and G2 (five consecutive days) groups (77.0 ± 39.4%) as compare to HF group (Supplementary Table 3) (Figure 2C). G-CSF treatment decreased the gene expression of triglyceride (SREBP1c, SCD-1, FAS), cholesterol (SREBP2, HMG-CoA reductase) biosynthesis and inflammation markers (TNF-α, MCP-1), in all G1, G2 and G3 groups as compared to the HF group (Figure 3A). Caspase-3 expression was also decreased in all G-CSF treatment groups compared to HF group (*p* < 0.05) (Figure 2D). The anti-apoptotic Bcl-2 protein expression declined in HF group compared to control, while increased in all G1, G2, and G3 groups. The apoptotic Bax protein expression increased in HF group, while decreased in all G-CSF treatment groups (Figure 3B).

### Effects of long acting G-CSF administration on MCD Diet induced NAFLD model

**Figure 4:**
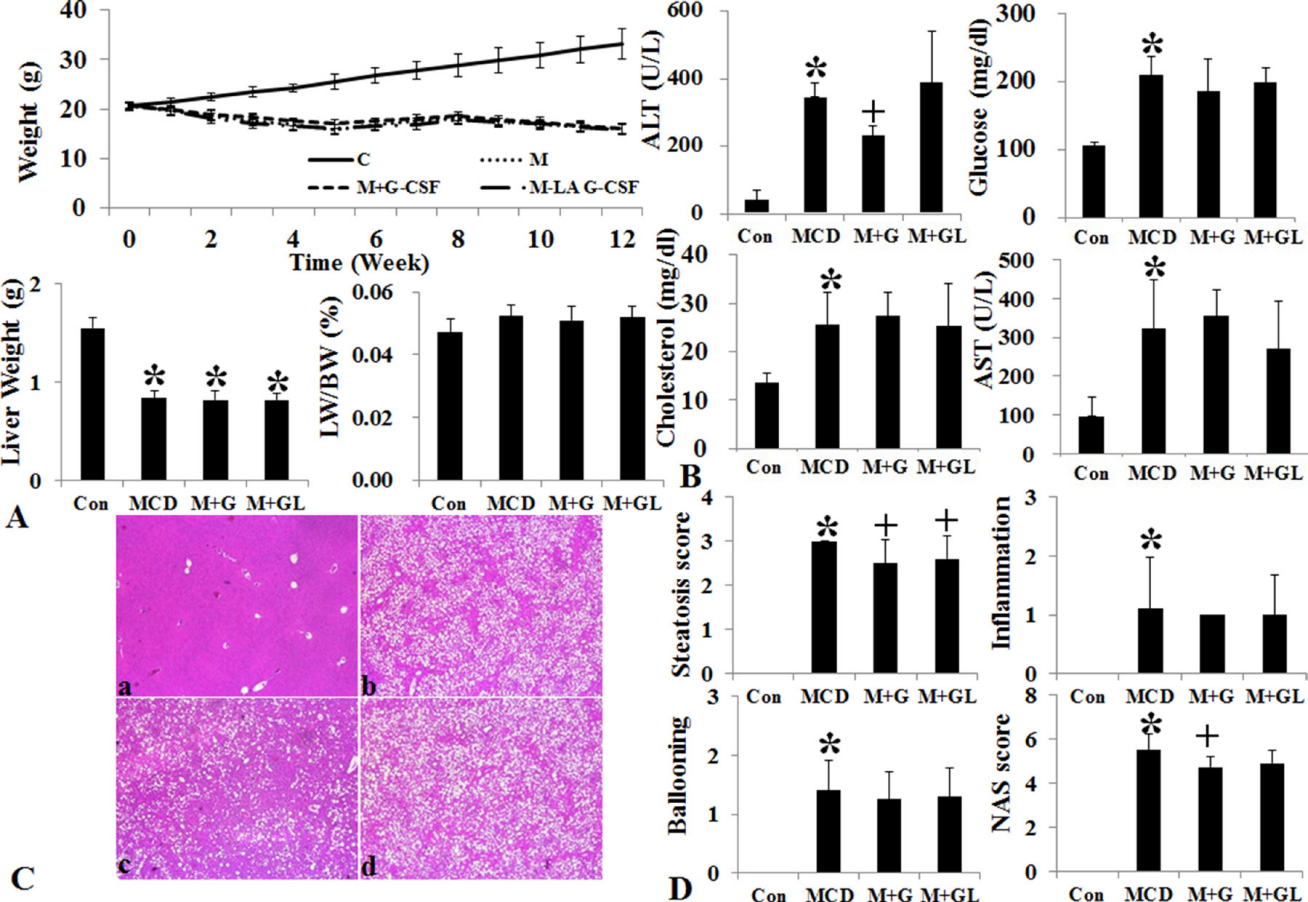
Changes of body weight, liver histology and biochemistry following long acting G-CSF treatment in methionine choline deficiency diet induced fatty liver model Body weight and liver weight and liver to body weight ratio of MCD group decreased as compared to control group (**A**). Serum ALT decreased in M+G group compared to MCD group (**B**). Hematoxylin & eosin staining results following G-CSF treatment in MCD diet fed groups. The steatosis score decreased in both M+G, M+GL groups as compared to MCD group, NAS (non-alcoholic fatty liver disease activity score) only decreased in M+G group as compared to the MCD group (**C** and **D**). Control group (*n* = 8), MCD (*n* = 8), M+G group (*n* = 10), M+GL group (*n* = 10) ^*^p < 0.05 by ANOVA with post-hoc Duncan, when it compare to control group (C), ^+^*p* < 0.05 by ANOVA with post-hoc Duncan, when it compare to NAFLD group (N). Abbreviations: Con, control; MCD, methionine choline deficiency diet induced fatty liver model; M+G group, short acting G-CSF 30 μg/kg twice a week from 9th to 12th week. M+GL, long acting G-CSF 30 μg/kg once only in 9th week.

**Figure 5:**
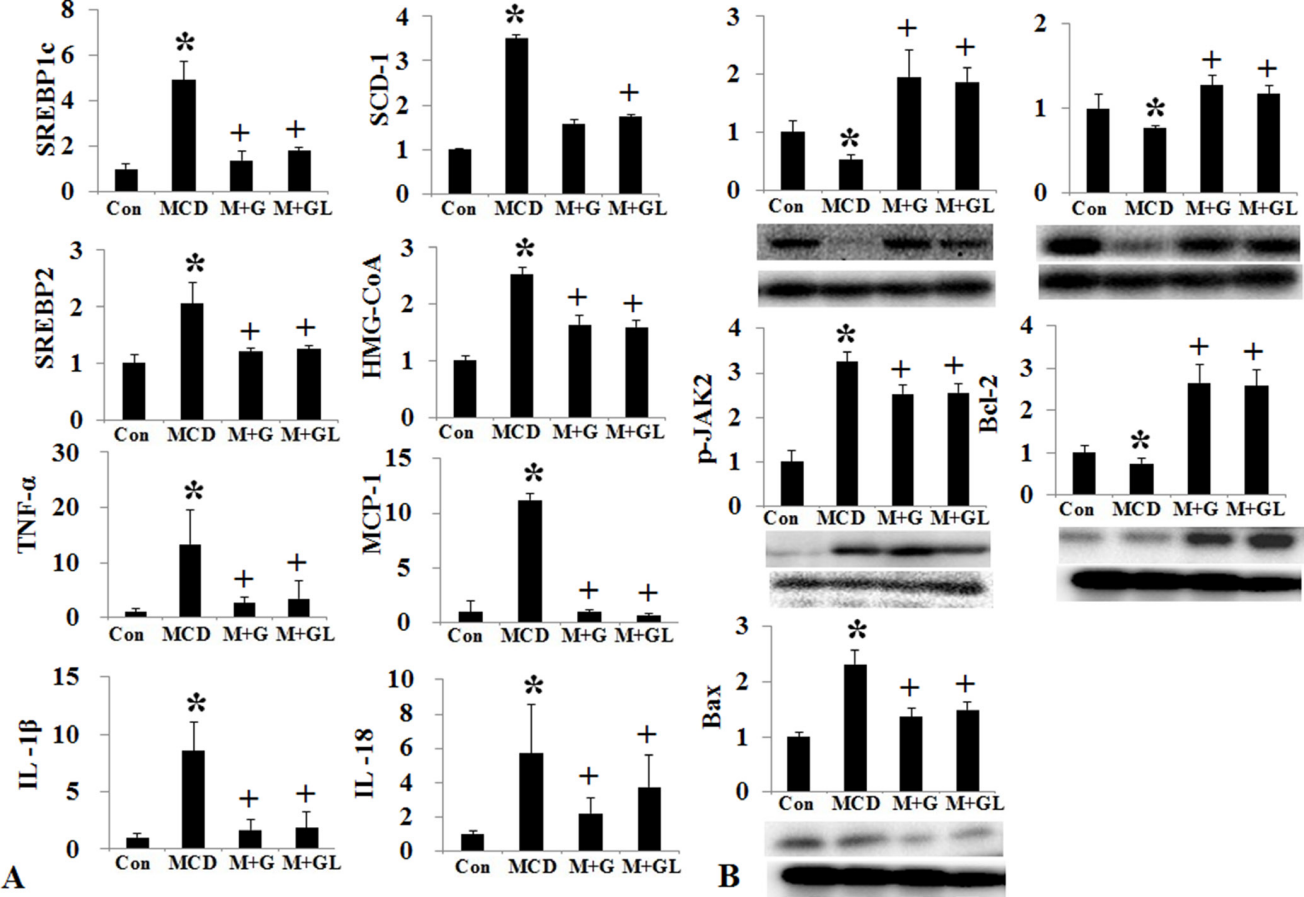
Changes of intrahepatic fat, cholesterol, inflammation, and apoptosis signal after long acting G-CSF treatment in methionine choline deficiency diet induced fatty liver model Triglyceride (SREBP1c, SCD-1, FAS), cholesterol de novo synthesis markers (SREBP2, HMG-CoA reductase), and intrahepatic inflammation decreased in long acting G-CSF treatment compare to control group (**A**). Change in cell proliferation and apoptosis in G-CSF treated MCD diet groups. G-CSF treatment (M+G, M+GL groups) increased Bcl-2 expression and decreased Bax expression as compared to MCD group (**B**). Control group (*n* = 8), MCD (*n* = 8), M+G group (*n* = 10), M+GL group (*n* = 10). ^*^p < 0.05 by ANOVA. Abbreviations: Con, control; MCD, methionine choline deficiency diet induced fatty liver model; M+G group, short acting G-CSF 30 μg/kg twice a week from 9th to 12th week. M+GL, long acting G-CSF 30 μg/kg once only in 9th week.

Conventional short acting G-CSF 30 μg/kg (twice a week for one month, M+G group) and long acting G-CSF (once in 9th week) 30 μg/kg was administered in MCD induced NAFLD model. The body weight, liver weight and liver to body weight ratio were not different among the NAFLD and G-CSF treatment groups (M+G, M+GL) (Supplementary Table 4) (Figure 4). ALT decreased in conventional G-CSF treatment (M+G) group as compared to the MCD group (Supplementary Table 5). Both the conventional G-CSF twice a week (M+G) and long acting G-CSF once in 9th week (M+GL) groups decreased hepatic steatosis score as compared to MCD group. The NAS score also decreased significantly in M+G group (*p* = 0.02), but not in M+GL groups. The expression of triglyceride (SREBP1c, SCD-1, FAS), cholesterol biosynthetic marker (SREBP2, HMG-CoA reductase) and inflammatory markers (TNF-α, MCP-1) also decreased in both conventional and long acting G-CSF groups compared to the MCD group (Figure 5A). Anti-apoptotic Bcl-2 protein expression increased while pro-apoptotic Bax protein expression decreased in the both G-CSF treatment groups as compared to MCD group (Figure 5B).

### Marrow cell mobilization according to various G-CSF protocols

**Figure 6:**
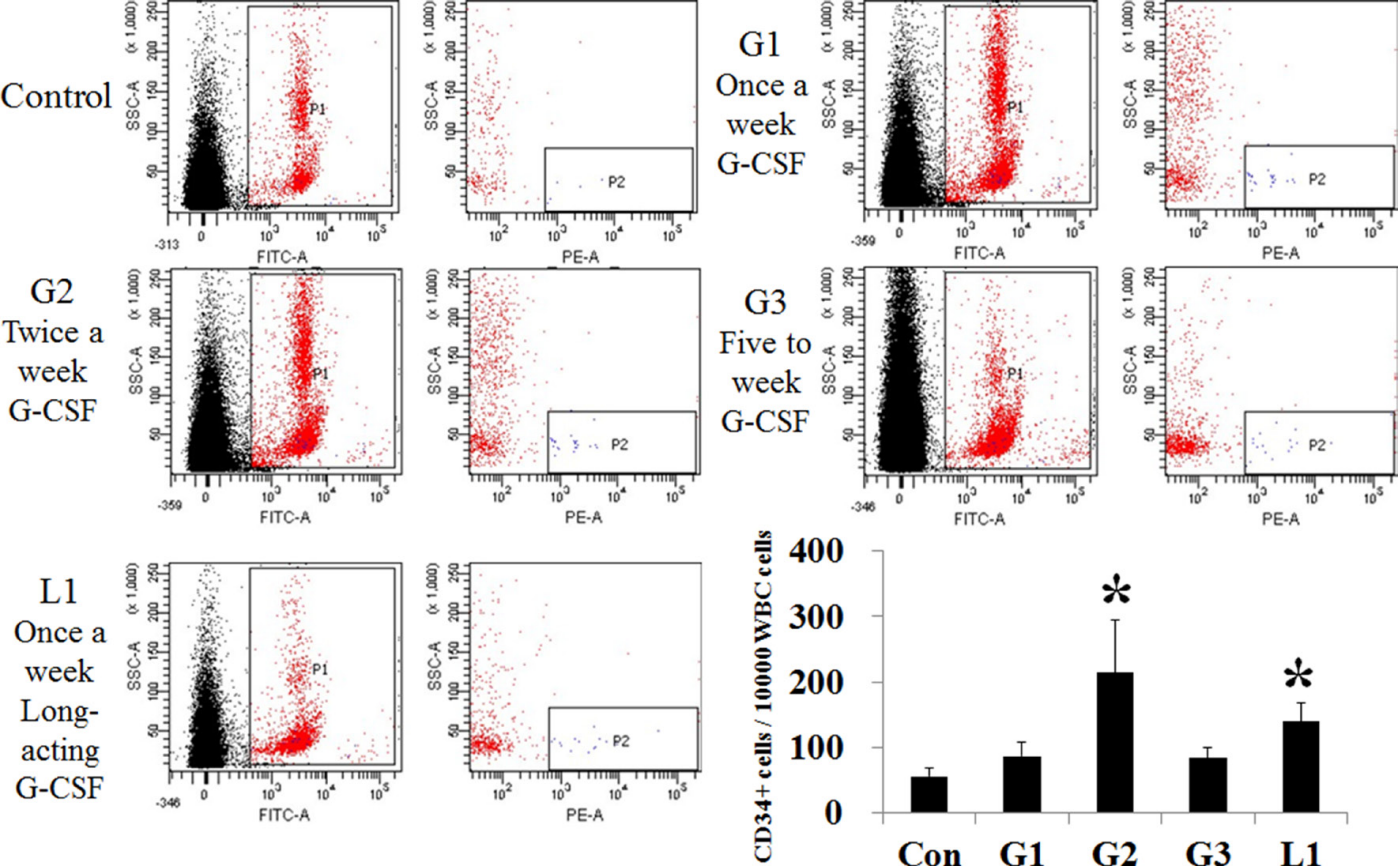
Comparison of stem cell mobilization after administration of hepatotropic dose of G-CSF The number of CD34+ positive cells increased in the G2 (*n* = 5) and L1 group (*n* = 5), compared to the control. G1 (*n* = 5) and hepatotropic dose G3 (*n* = 5) groups had no difference from the control. ^*^p < 0.05 by ANOVA with post-hoc Duncan, when it compare to control group (C), ^+^*p* < 0.05 by ANOVA with post-hoc Duncan, when it compare to NAFLD group (N). Abbreviations: Con, control; G1, G-CSF treatment once weekly; G2, G-CSF treatment daily for 5 consecutive days; G3, G-CSF treatment twice weekly; L1, long acting G-CSF once.

Bone marrow stem cell mobilization was assessed by number of CD34+ cell among peripheral leukocyte using flow cytometry (Figure 6). The number of CD34+ cells, out of 10,000 CD45+ cells, increased in the conventional G-CSF five consecutive days (G2 group) and the long acting G-CSF group (L1, long acting G-CSF once a month) compared to the control group. But conventional G-CSF once a week (G1) and twice a week (G3) protocol did not increase number of peripheral CD34+ cells.

### Hepatotropic effects of G-CSF via PI3K-Akt activation

**Figure 7:**
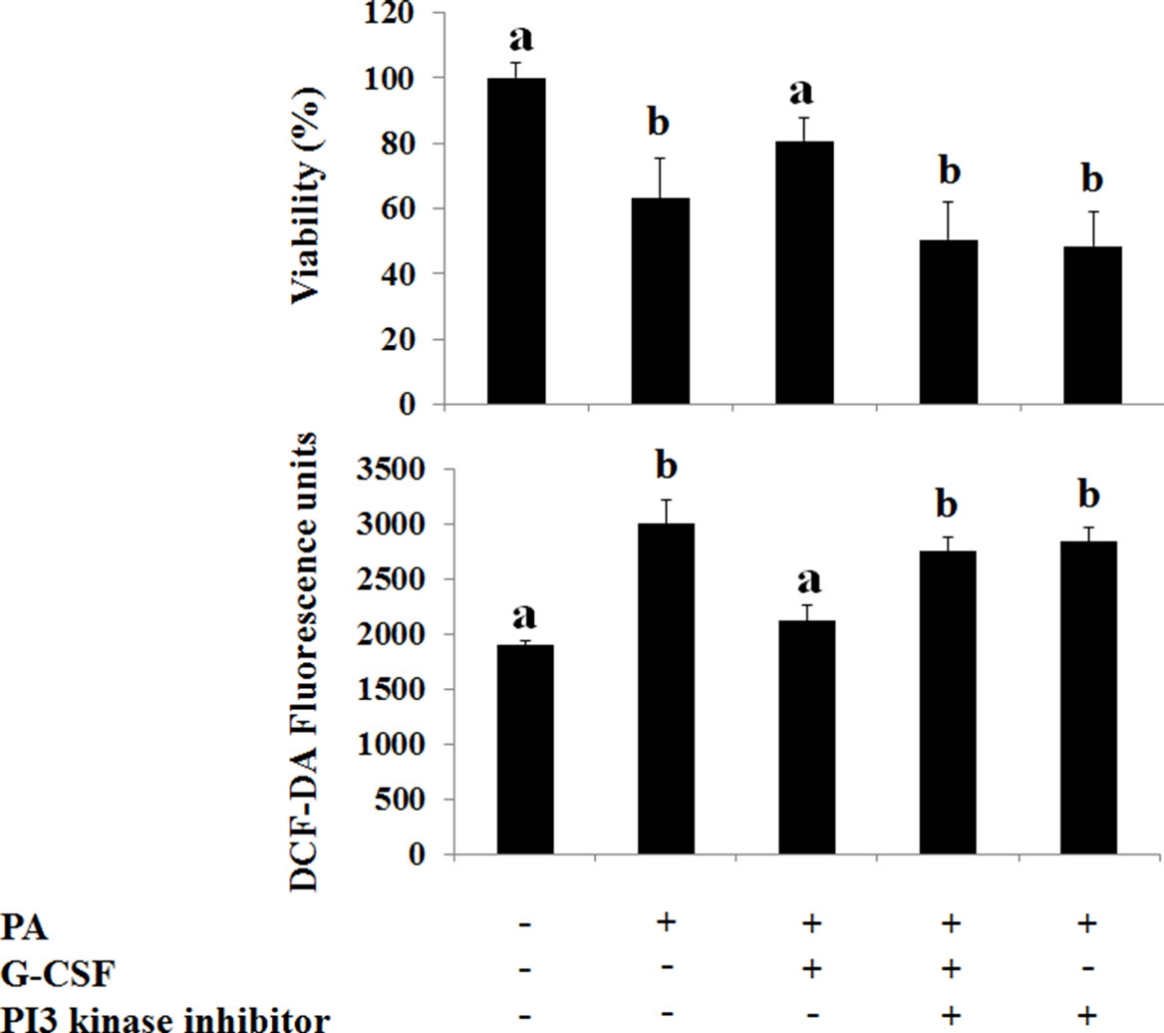
Effect of G-CSF and G-CSF inhibitor on cell viability, oxidative stress, G-CSFr expression on *In Vitro* lipotoxicity models G-CSF treatment increased viability as compared to PA group. G-CSF+PI-3 kinase inhibitor co-treatment blocked protective effects of G-CSF. DCF-DA, reactive oxygen species marker (ROS), increased in PA group as compared to the control group. G-CSF treatment decreased ROS as compared to PA group. G-CSF+PI-3 kinase inhibitor co-treatment increased ROS as compared to G-CSF group. ^*^p < 0.05 by ANOVA with post-hoc Duncan, when it compare to control group (C), ^+^*p* < 0.05 by ANOVA with post-hoc Duncan, when it compare to NAFLD group (N). Abbreviations: PA, Palmitic acid.

*The p-*PI3 kinase and *p*-Akt expression decreased in both HF and MCD induced NAFLD models (Figures 3B, 5B). The conventional G-CSF and long acting G-CSF restored *p*-PI3 kinase and *p*-Akt expression in both HF and MCD induced NAFLD models. The role of *p*-PI3 kinase was reconfirmed using *In vitro* NAFLD model. Following PA treatment, the HepG2 cells showed decreased viability which was improved following G-CSF treatment (Figure 7). To further confirm whether it is PI3 kinase which is involved in G-CSF mediated improved viability, the PA treated HepG2 cells were co-treated with G-CSF and PI3 kinase inhibitor. HepG2 cells co-treated with G-CSF and PI3 kinase inhibitor did not show improved cell viability. Similarly, co-treated cells failed to reduce ROS production, while G-CSF mono-treatment showed decreased ROS in *In vitro* lipotoxicity model (Figure 7).

### Hepatotropic effects of G-CSF *in vitro* lipotoxicity model

**Figure 8:**
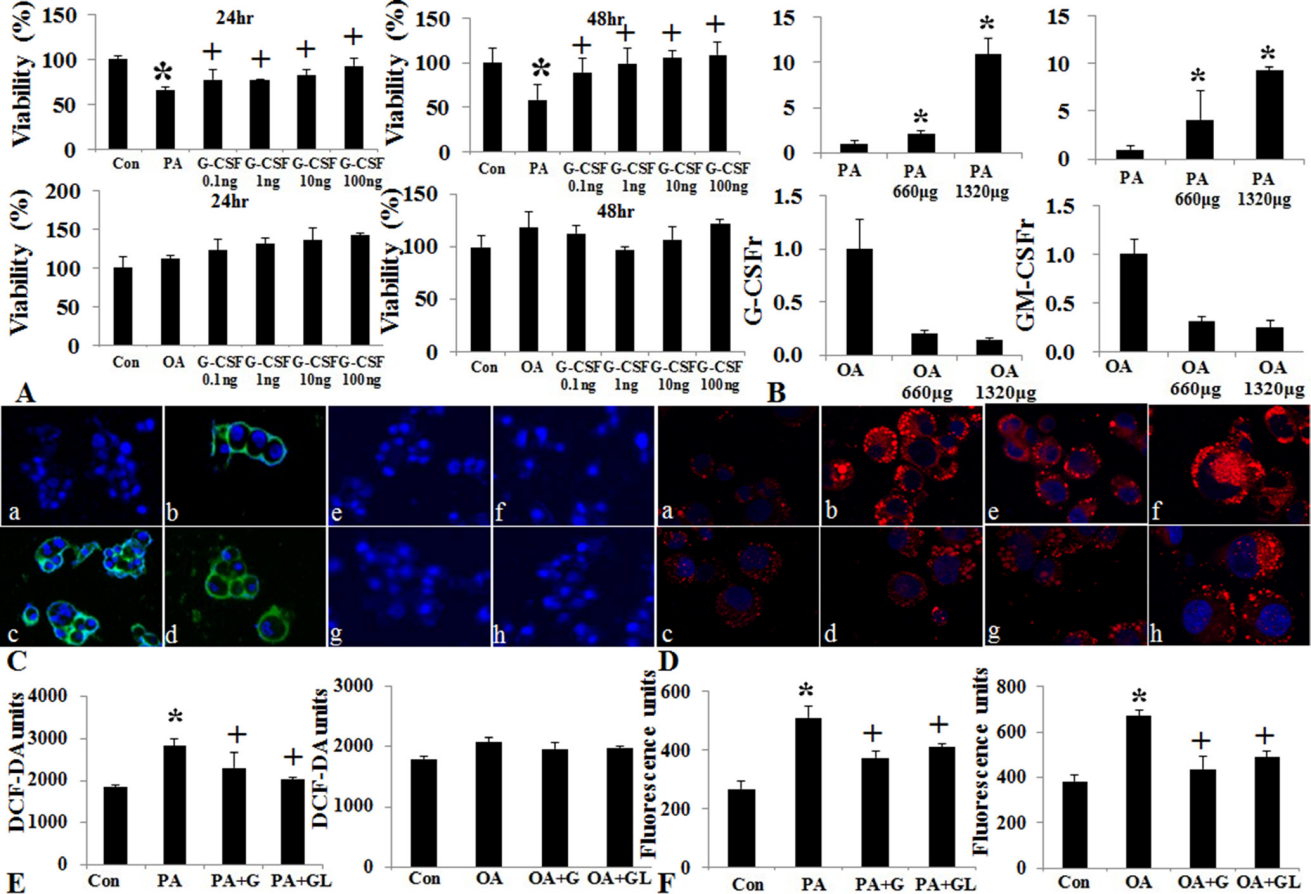
Effect of G-CSF on cell viability, oxidative stress, and G-CSFr expression in PA and OA induced vitro lipotoxicity models Cell viability decreased following PA treatment, G-CSF administration increased cell viability in both 24 and 48 hr treatment groups (**A**). Change in G-CSFr and GM-CSFr Expression following PA and OA lipotoxicity models. G-CSFr and GM-CSFr expression increased in PA not OA treatment (**B**). Immunofluorescence stain of G-CSFr following PA and OA treatment. GM-CSFr expression increased in PA group as compared to control group (a: control, b: PA treatment, c: PA + G-CSF, d: PA + long acting G-CSF, e: control, f: OA, g: OA + G-CSF, h: OA + long acting G-CSF) (**C**). Nile red O staining for triglyceride following PA and OA Pre-treatment. G-CSF treatment decreased the staining in both PA and OA groups (**D** and **F**). ROS Expression following PA and OA Pre-treatment. G-CSF treatment decreased ROS as compared to PA and OA groups (**E**). a and b *p* < 0.05 by ANOVA with post-hoc Duncan. Abbreviations: PA, Palmitic acid; OA, Oleic acid; ROS, reactive oxygen species.

The HepG2 cell viability decreased following saturated fatty acid (PA) treatment but not following unsaturated fatty acid (OA) treatment (Figure 8A). The G-CSFr expression increased according to PA concentration, but not following OA concentration (Figure 8B and 8C). G-CSF treatment restored cell viability in PA induced lipotoxicity but not following OA treatment. Both the PA and OA treatment showed increased intracellular fat deposition as compared to control, while G-CSF treatment decreased intracellular triglyceride contents (Figure 8D). The ROS assay indicated that saturated fatty acid (PA) increased ROS production as compared to control (*p* = 0.01). Both conventional G-CSF and long-acting G-CSF treatment decreased ROS production as compared to PA induced lipotoxicity (Figure 8E). However, these protective effects of G-CSF treatment were not observed with O.A induced lipotoxicity.

### G-CSF receptor expression in human NAFLD patients

**Figure 9:**
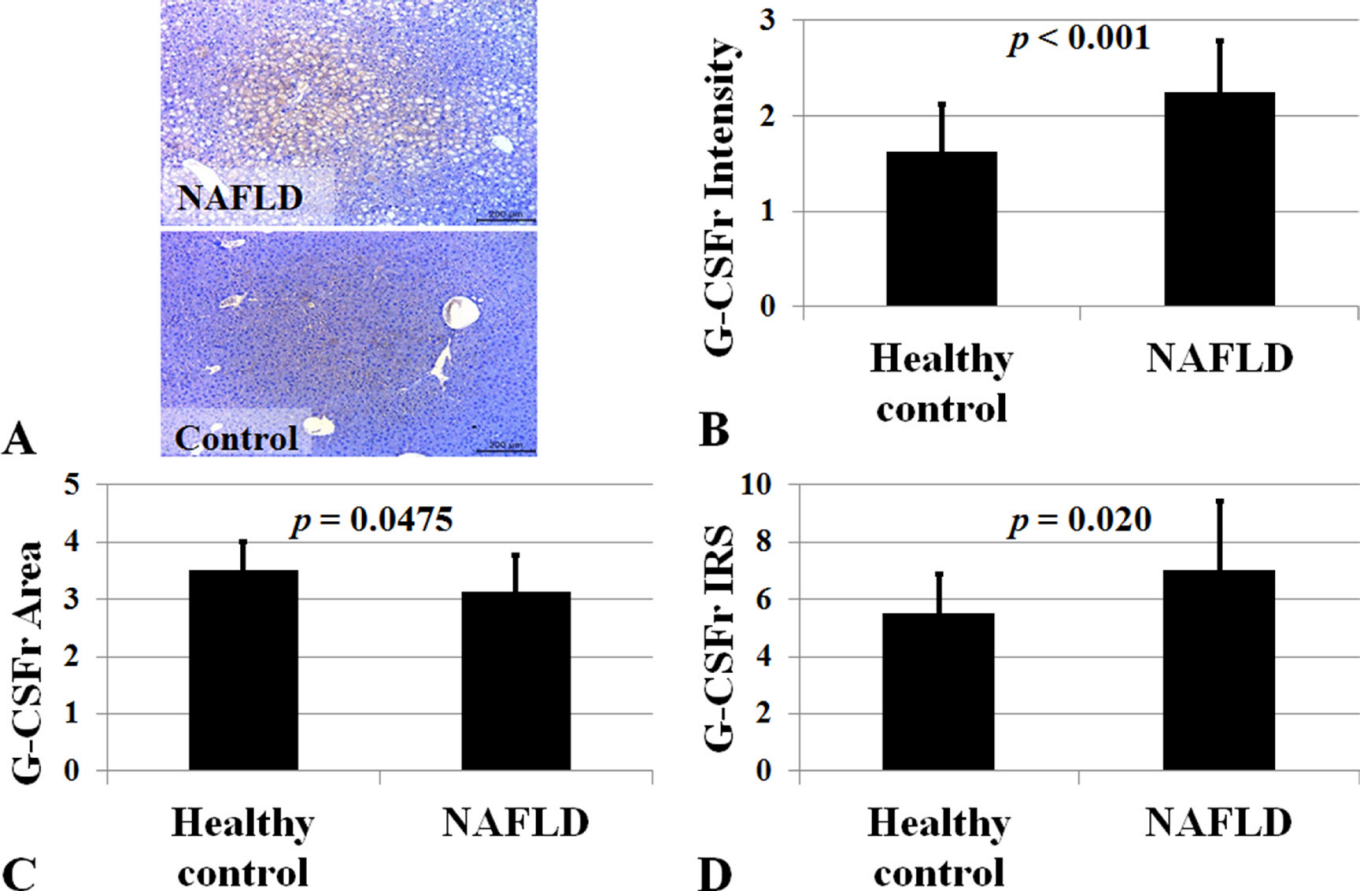
G-CSF receptor expression in NAFLD subjects Immune reactive score (IRS) for G-CSF receptor was higher in NAFLD group (*n* = 37) than healthy control group (*n* = 16). *P* < 0.05 by Mann-Whitney *U* test. G-CSF receptor expression was higher in NAFLD (**A**). Intensity, positive cell proportion, and immune reactive score (IRS) increased in NAFLD (**B**–**D**). IRS was calculated by multiplication of positive cells proportion and staining intensity.

We studied 37 NAFLD and 16 control subjects. G-CSF receptor intensity was higher in NAFLD group than healthy control group (2.24 ± 0.54 vs. 1.62 ± 0.50, *p* < 0.001) (Figure 9). Extent of G-CSF receptor satin was higher in healthy control group than NAFLD group (3.50 ± 0.51 vs. 3.13 ± 0.63, *p* = 0.047). Immune reactive score (IRS) was higher in NAFLD group than healthy control group (7.05 ± 2.41 vs. 5.50 ± 1.36, *p* < 0.001).

## DISCUSSION

The current study found that G-CSFr is normally expressed at very low levels in normal hepatocytes and is increased following hepatic injury. Hepatotropic dose (twice a week) G-CSF without marrow stem cell mobilization improves liver histology and cell survival via PI3K/Akt activation.

Our data showed that G-CSFr increased more than five times in NAFLD, alcoholic hepatitis, and hepatic IR model except cirrhosis model (Figure 1). NAFLD, alcoholic hepatitis, and hepatic IR model share hepatocyte apoptosis as crucial a pathophysiology. Moreover, PA induced lipotoxicity model, but not OA induced simple steatosis increased G-CSFr. These results re-confirmed the G-CSFr expressed in hepatocyte might be strongly associated with hepatocyte apoptosis. It suggested that ‘hepatocyte G-CSFr’ can be a potential target for steatohepatitis treatment.

Most G-CSF studies have focused on stem cell mobilization to target organ as mode of action. All most all of previous studies used repeated high-dose injection (five day consecutive injection), because it has been believed that bone marrow stem cell mobilization and homing to target organ is the key mechanism. Our data showed hepatotropic doses G-CSF (twice a week) decreased hepatic inflammation and cell death without marrow cell mobilization. FACS analysis showed increased marrow cell mobilization in peripheral blood following five times a week G-CSF (30 μg/kg) and long acting G-CSF (30 μg/kg) once a month administration. However, marrow cell mobilization was not observed with twice a week conventional G-CSF (30 μg/kg) administration. Provided that steatohepatitis is a chronic disease, low-dose G-CSF administration is more practically appropriate than repeated high-dose injection, which might lead to complications such as splenetic rupture in normal individuals [10]. Although once a month 30 μg/kg long acting G-CSF treatment increased marrow cell mobilization, less than 30 μg/kg long acting protocol might be promising.

**Figure 10:**
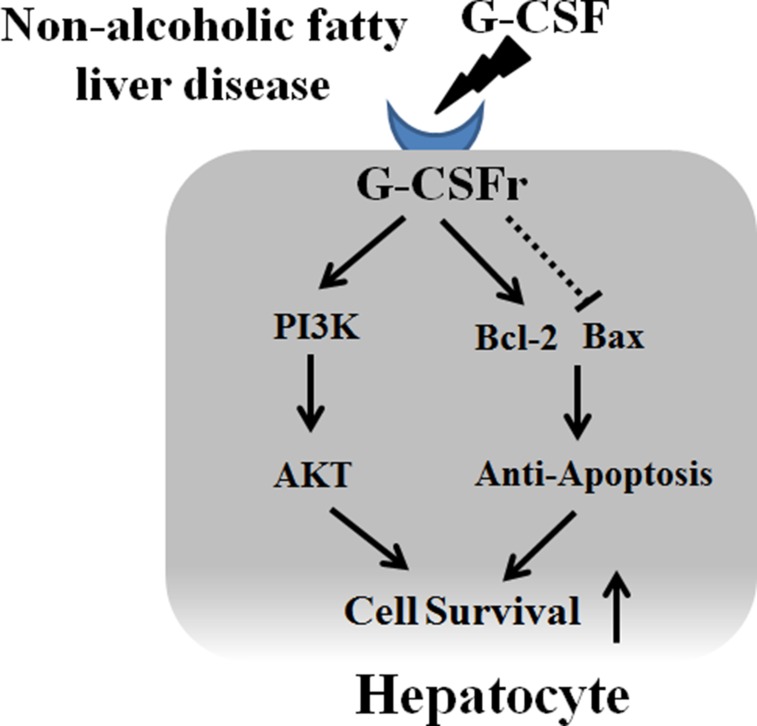
Proposed mechanism of G-CSF in hepatocyte G-CSF receptor expression in normal hepatocyte and receptor expression increased in NAFLD patients. G-CSF increased PI3K/Akt expression in hepatocyte as well as decreased apoptotic drive (increased Bcl-2 expression and decreased Bax expression).

To the best of our knowledge, this is the first study to document the direct hepatotrophic effects of G-CSF via PI3K/Akt activation. Our data clearly demonstrated G-CSF treatment increased cell survival, and anti-inflammatory effects against lipotoxicity. G-CSF receptor expression in normal hepatocyte and receptor expression increased in NAFLD patients compare to control group (Figure 9). G-CSF treatment increased PI3K/Akt expression in hepatocyte as well as decreased apoptotic drive (increased Bcl-2 expression and decreased Bax expression) (Figure 10). Protective effects of G-CSF on NAFLD mediated via PI3K/Akt pathway. PI3K inhibitor masked the G-CSF induced hepatocytes protection in lipotoxicity models. PI3K/Akt/mTOR pathway plays an important role in cell growth, cell survival control, metabolism and apoptosis. But organ protective effects of G-CSF appeared to vary depending on organs and disease models. For instance, G-CSF protects myocardium by activating JAK/STAT3 pathway rather than PI3K/Akt pathway [11, 12]. G-CSF showed neuronal trophic effects via activating JAK/STAT5 and Bcl-2 [13]. Some studies also report G-CSF activates PI3K/Akt, MEK and ERK pathways in lung cancer cells [14]. G-CSF seems to exert its protective effects via different pathways in diverse organ and disease models.

There were several limitations in this study. First, our data showed that hepatotropic dose G-CSF (twice a week) could improve inflammation and hepatocyte apoptosis in NAFLD for the first time, but the effects of G-CSF seem not so different in each group G1 (once a week), G2 (five days), and G3 (twice a week), and there was only a small effect. Second, although G-CSF treatment reduced intracellular fat deposition in *In Vitro* model, G-CSF treatment decreased hepatic fat accumulation in only MCD induced steatohepatitis model, but improving steatosis was not observed in HF induced model. Effects of G-CSF on steatosis seemed to be somewhat complex and some other mechanism might be involved beyond G-CSFr pathway. Third, marrow cell mobilization by G-CSF treatment was evaluated in healthy mouse. Because expression of G-CSFr increases in disease models, it can't be justified in disease models as well.

In conclusion, G-CSFr expressed in normal hepatocyte with low level, and hepatic G-CSFr expression increased in NAFLD. The hepatotropic dose of G-CSF improves hepatic inflammation and cell survival via PI3K/Akt activation.

## MATERIALS AND METHODS

### Hepatic G-CSF receptor expression in various liver disease models

Hepatic G-CSF receptor (G-CSFr) and GM-CSF receptor (GM-CSFr) expression was evaluated in five different liver disease models to evaluate the possibility of hepatotropic effects of G-CSF. The liver disease models were established as follow; Non-alcoholic fatty liver disease model: feeding high-fat (HF) diet (60%kcal) for 12 weeks (*n* = 10) [15], Alcoholic hepatitis model: feeding Lieber–DeCarli alcohol diet containing 6% ethanol (Alcohol) for 10 weeks (*n* = 10) [16], Acute toxic hepatitis model: administration of 200 mg/kg thioacetamide (TAA) for three consecutive days by peritoneal injection (A-TAA) (*n* = 10) [17], Cirrhosis: administration of 100 mg/kg TAA twice a week for 4 weeks by peritoneal injection (Ch-TAA) (*n* = 10) [17], Hepatic ischemic reperfusion (IR) injury: clamping of hepatic artery and portal vein for one hour followed by reperfusion for four hours) (*n* = 10) [18].

### Conventional G-CSF treatment in NAFLD

Six-week old male C57BL/6J mice (*n* = 46) (Orient Animal Laboratory, Seoul, South Korea) were randomly divided into control (C), NAFLD (N), and three kinds of G-CSF treatment groups (G1–G3). Control group (*n* = 8) was fed normal chow while NAFLD (*n* = 8) and three G-CSF treatment groups were fed high fat (HF) diet (60%kcal) for 12 weeks. G-CSF groups received conventional short acting G-CSF 30 μg/kg (Leucostim^®^, Dong A Pharmaceutical Co., Ltd, Seoul, South Korea) i.p dissolved in 100 μL sterile saline such as three kinds of protocols: G1 (*n* = 10), once weekly from 8th to 12th week; G2 (*n* = 10), once daily for 5 consecutive days in 9th week; G3 (*n* = 10), twice weekly (three days apart) from 9th to 12th week (Supplementary Figure 1). After 12 weeks the animals were euthanized and blood and liver samples were obtained for further analysis.

### Long acting G-CSF treatment in NAFLD

Six-week old male C57BL/6J mice (*n* = 36) (Orient Animal Laboratory, Seoul, South Korea) were randomly divided control (C), MCD and two types of G-CSF treatment groups (M+G and M+GL). Control group (*n* = 8) was fed normal chow while MCD (*n* = 8) and G-CSF groups were fed MCD diet for 12 weeks. M+G group (*n* = 10) received short acting G-CSF 30 μg/kg (Leucostim^®^, Dong A Pharmaceutical Co., Ltd, Seoul, South Korea), i.p dissolved in 100 μL sterile saline twice weekly from 9th to 12th week. M+GL group (*n* = 10) received long acting G-CSF 30 μg/kg (HM10460A, Hanmi Pharm. Co, Ltd., Seoul, South Korea) i.p dissolved in 100 μL sterile saline once only in 9th week (Supplementary Figure 2). After 12 weeks the animals were euthanized and blood and liver samples were collected for further analysis. The mice were kept in a controlled lab environment in which the temperature and humidity were maintained at 23 ± 2°C and 60 ± 10% under a 12-hour light/12-hour dark cycle. The animals had free access to water and feed. All the experimental procedures were approved by the Institutional Animal Care and Use Committee of Hanyang University (HY-IACUC-2014-0215).

### Human liver sample and interpretation

Liver biopsy samples were obtained from 37 NAFLD subjects and 16 normal healthy controls. Patients with HBsAg positive, HCV antibody positive, drug-induced hepatitis, alcohol consumption of > 210 g/week in men and > 140 g/week in women were also excluded. Healthy normal liver tissue obtained from trauma operation. The study was approved by the institutional review board at Hanyang University Hospital (2017-03-002). G-CSF receptor immunostaining was expressed by intensity, extent, and immune reactive score (IRS). IRS was calculated by multiplying the intensity score by proportion score (extent). An intensity score was assigned from 0 to 3 points. The extent score was given from 0 to 4 points.

### Serum analysis

The blood was withdrawn from the heart was centrifuged at 4°C and 3,000 rpm for 10 minutes to obtain serum. The serum aspartate transaminase (AST), alanine transaminase (ALT), glucose, triglyceride and cholesterol were measures using biochemical analytical system (Hitachi-747; Hitachi, Tokyo, Japan).

### Histological analysis

Paraformaldehyde fixed paraffin embedded liver tissues were sectioned (4μm), H&E staining was performed and was evaluated using optical microscope. Hepatic steatosis was scored as follows; 5% (Score, 0); 5–33% (Score, 1), > 33–66% (Score, 2) and > 66% (Score, 3); 0–3; lobular inflammation, 0–4; periportal activation, 0–4; and fibrosis, 0–4 [19]. For immunohistochemistry, the live sections fixed on slides were heated in the microwave at 92–98°C for 15 minutes using a 0.01M antigen retrieval buffer. Further, endogenous peroxidase activity was quenched with 0.3% hydrogen peroxide. Hepatocyte apoptosis was evaluated by the Caspase-3 (Santa Cruz, CA, USA) antibody.

### Stem cell mobilization analysis

4C57BL/6 mice (*n* = 25) (Orient Animal Laboratory, Seoul, South Korea) were randomly divided into 5 groups; Control (Con) (*n* = 5); G1 (*n* = 5), 30 μg/kg G-CSF once a week by intraperitoneal injection; G2 (*n* = 5), 30 μg/kg 5 times a week; G3 (*n* = 5), 30 μg/kg twice a week; L1 (*n* = 5), 30 μg/kg long acting G-CSF once. After one week 200 μl blood was collected through heart puncture, and after 24 hours of last G-CSF injection. RBCs were removed using red blood cell (RBC) lysis buffer (Sigma, CA, USA) and centrifuged at 3,500 rpm for 10 min. 10 μl phycoerythrin-conjugated anti-CD34 (CD34-PE, clone 581) and fluorescein isothiocyanate–conjugated anti-CD45 (BD Bioscience, CA, USA) were added per sample, and 7-AAD (BD Bioscience, CA, USA) helped identifying live cells. After staining, 4% paraformaldehyde was added for 10 minutes to fix cells. Further, 300 μl PBS was added for measurement. Fluorescence-activated cell sorting (FACS) canto (BD Bioscience, CA, USA) FACS DivaTM were used as FACS M/C and S/W, respectively.

### Statistics

All the experimental values are expressed as average ± standard error and were obtained after three independent replicates. SPSS statistics 21 (SPSS Inc., Chicago, IL, USA) was used for statistical analysis. The measured values and significance of control and G-CSF groups were determined using ANOVA. *p* < 0.05 was considered statistically significant.

## SUPPLEMENTARY MATERIALS FIGURES AND TABLES



## Abbreviations

NAFLD: Non-alcoholic fatty liver disease
G-CSF: Granulocyte-colony stimulating factor
GM-CSF: Granulocyte Macrophage-colony stimulating factor
HF: High Fat
MCD: Methionine and choline deficient
A-TAA: Acute thioacetamide induced toxic hepatitis model
Ch-TAA: Chronic thioacetamide induced cirrhosis model
IR: Ischemic reperfusion
BW: Body weight
LW: Liver weight
ALT: Alanine aminotransferase
AST: Aspartate aminotransferase
SREBP1c: Sterol regulatory element-binding protein
FAS: Fatty acid synthase
SCD-1: Stearoyl-CoA desaturase-1
SREBP2: Sterol regulatory element-binding protein 2
HMG-CoA reductase: 3-hydroxy-3-methyl-glutaryl-CoA reductase
MCP-1: Monocyte chemotactic protein 1
TNF-α: Tumor necrosis factor alpha
IL-1β: Interleukin-1 beta
IL-18: Interleukin-18
BCL-2: B-cell lymphoma 2
BAX: BCL2-associated X protein
PI3 kinase: Phosphoinositide 3 kinase
JAK2: Janus-activated kinase 2
Akt: Protein kinase B
MTT: 3-(4,5-Dimethylthiazol-2-yl)-2,5-diphenyltetrazolium bromide, a tetrazole
ROS: Reactive oxygen species
