# Supplementary material for: Granulocyte colony stimulating factor treatment in non-alcoholic fatty liver disease: beyond marrow cell mobilization
Source: Oncotarget. 2017 Jul 4;8(58):97965–76. doi: 10.18632/oncotarget.18967 (PMC5716706; doi:10.18632/oncotarget.18967)
Supplement: Supplementary file 1 [file oncotarget-08-97965-s001.pdf]

# Granulocyte colony stimulating factor treatment in non-alcoholic fatty liver disease: beyond marrow cell mobilization

## SUPPLEMENTARY MATERIALS

## SUPPLEMENTARY MATERIALS AND METHODS

### RNA acquisition, cDNA synthesis and real-time PCR analysis

The tissues were homogenized with 1ml Trizol Reagent (Invitrogen, CA, USA) per 200 mg tissue. 0.2 ml chloroform per 1 ml Trizol was added and samples were centrifugation at 12,000 rpm for 10 minutes. The supernatant was collected and 500 µl isopropanol was added to it. The samples were later centrifuged at 12,000 rpm and 4°C for 10 minutes. 1 ml 75% ethanol was added to precipitate mRNA and was further centrifuged at 4°C and 12,000 rpm for 10 minutes followed by washing, drying, dissolving in 200 µl DEPC water. The RNA was stored at –70°C. The absorbance was measured by UV spectrophotometer (Beckman DU730) at 260–280 nm to confirm whether mRNA purity was between 1.9 and 2.1 for quantification. cDNA was synthesized using Maxime RT PreMix (iNtRon, Gyeonggi, Korea) by adding 1 µg mRNA, primer, and DEPC water. Real-time PCR utilized 2 µg cDNA using LC480 (Roche, IN, USA). 10X PCR buffer (Takara-Bio, Kusatsu, Japan), dNTP (Takara-Bio, Kusatsu, Japan), SYBR Green (Roche, IN, USA) and primer (Bioneer, Seoul, Korea) were also added. Real-time PCR was repeatedly carried out 35 times each for 10 seconds at 95°C, 59°C and 72°C and set at 4°C. Supplementary Table 6 shows the primer information for SRRBP1c, FAS, SCD-1, SREBP2, HMG-CoA reductase, TNF-a, MCP1, G-CSFr and GM-CSFr.

### Protein extraction and western blot analysis

The tissues were homogenized after adding 1ml PRO-PREP™ (iNtRon, Gyeonggi, Korea) to 20mg tissue and was kept on ice for about 30 minutes for cytolysis and centrifuged at 12,000 rpm and 4°C for 15 minutes to generate supernatant. Protein concentration was measured using Bradford assay (Molecular devices max190). For Western blot, 30–60 µg protein per well was loaded on 10% sodium dodecylsulfate–plyacrylamide gel for electrophoresis (SDS–PAGE). The proteins were transferred on PVDF membrane (Amersham, London, UK). The membrane was incubated with primary antibody for overnight at 4°C. The membrane was later incubated with secondary antibody and protein expression was confirmed using ECL (GenDEPOT, Seoul, Korea). The antibodies used were as follows: Bax (Santa Cruz, CA, USA), Bcl-2 (Cell Signaling Technology, MA, USA), GAPDH (Cell Signaling Technology, MA, USA), PI3 kinase (Cell Signaling, USA, 4292), p-PI3 kinase (Cell Signaling Technology, MA, USA), JAK2 (Cell Signaling Technology, MA, USA), p-JAK2 (Cell Signaling Technology, MA, USA), Akt (Cell Signaling Technology, MA, USA), and p-Akt (Cell Signaling Technology, MA, USA). The expression intensity of blots was measured using Image Lab 3.0 software (Bio-Rad).

### Cell culture

Human hepatoma cell line (HepG2) were cultured in Dulbecco's modified Eagle's medium (Gibco, NY, USA) containing fetal bovine serum (Gibco, NY, USA) and 100 mg/mL streptomycin (Gibco, NY, USA) using 5% $CO_2$ incubator at 37°C.

### Assessment of cell viability (MTT Assay)

1.5 × $10^5$ cells were seeded on a 96 well plate. After 24 hour 400 µM PA and 800 µM OA was added to establish vitro NAFLD model. After 2 hr G-CSF (0.1–100 ng/ml) was added to the wells. After 24 and 48 hours, 20 µl Cell Titer 96 Aqueous One Solution (Promega, Madison, WI, USA) was added to determine cell viability. After 4 hr, 96-well plate was read using Microplate Reader (Emax microplate reader, Molecular Devices, CA, USA) at 490 nm.

### Assessment of oxidative stress (ROS Assay)

1.5 × $10^5$ cells were seeded on 96 well plate. After 24 hour 400 µM PA and 800 µM OA was added to establish vitro NAFLD model. After 2 hour, 100ng/ml G-CSF was added. After 24 hour 5 µM DCF-DA (Sigma, MO, USA) was added and reactive Oxygen Species (ROS) was measured using Victor3 (Perkin Elmer, MD, USA).

### Triglyceride measurements (Nile red Staining)

*In vitro* NAFLD model was established as previously mentioned. After 20 mins the cells were washed using PBS, 10 µg/ml Nile red (Sigma, CA, USA) was added and plate was read using Microplate reader (iMark, Bio-Rad, CA, USA).

**Supplementary Table 1: Body weight and liver weight, liver weight/body weight (LW/BW) changes according to treatment groups of high fat diet**

| Groups | 0 week (g) | 12th week (g) | *p* value | Liver weight (g) | *p* value | LW /BW (%) | *p* value |
|---|---|---|---|---|---|---|---|
| C | 19.1 ± 0.7 | $28.5 \pm 2.9^{+}$ | < 0.001 | $1.3 \pm 0.1^{+}$ | < 0.001 | 4.6 ± 0.6 | 0.882 |
| N | 19.1 ± 0.7 | $45.8 \pm 4.1^{*}$ | < 0.001 | $2.3 \pm 0.3^{*}$ | < 0.001 | 4.9 ± 0.4 | 0.882 |
| G1 | 19.0 ± 0.8 | $46.1 \pm 3.6^{*}$ | 1 | $2.0 \pm 0.5^{*}$ | 0.716 | 4.4 ± 1.0 | 0.526 |
| G2 | 19.4 ± 0.3 | $46.2 \pm 3.4^{*}$ | 0.999 | $1.9 \pm 0.4^{*}$ | 0.487 | 4.2 ± 0.7 | 0.280 |
| G3 | 19.1 ± 0.8 | $44.2 \pm 4.1^{*}$ | 0.885 | $1.5 \pm 0.5^{+}$ | 0.002 | $3.3 \pm 0.9^{*+}$ | 0.010 |

C, control; N, high fat induced fatty liver disease; G1, G-CSF treatment once weekly from 8th to 12th week; G2, G-CSF treatment daily for 5 consecutive days in 9th week; G3, G-CSF treatment twice weekly from 9th to 12th week. $^{*}p < 0.05$ versus control group. $^{+}p < 0.05$ versus High Fat diet group.

**Supplementary Table 2: Biochemical parameters of according to treatment groups of high fat diet**

| Groups | ALT (U/L) | AST (U/L) | T-cholesterol (mg/dl) | Glucose (mg/dl) | Triglyceride (mg/dl) |
|---|---|---|---|---|---|
| C | $31.0 \pm 4.1^{+}$ | 75.9 ± 13.1 | 71.2 ± 3.4 | 474.8 ± 47.1 | 31.0 ± 11.8 |
| N | $111.2 \pm 37.0^{*}$ | 106.2 ± 29.0 | $152.6 \pm 25.3^{*}$ | $543.6 \pm 31.0^{*}$ | $52.0 \pm 10.4^{*}$ |
| G1 | $68 \pm 25.5^{+}$ | 145.8 ± 50.3 | 137.0 ± 17.7 | 546.0 ± 36.0 | 66.0 ± 20.9 |
| G2 | $43.2 \pm 14.3^{+}$ | 114.0 ± 32.7 | 154.9 ± 9.6 | 557.0 ± 51.7 | 57.3 ± 25.8 |
| G3 | $32.3 \pm 10.3^{+}$ | 114.6 ± 48.6 | $107.5 \pm 30.1^{+}$ | $407.8 \pm 73.8^{+}$ | $30.1 \pm 12.1^{+}$ |

C, control; N, high fat induced fatty liver disease; G1, G-CSF treatment once weekly from 8th to 12th week; G2, G-CSF treatment daily for 5 consecutive days in 9th week; G3, G-CSF treatment twice weekly from 9th to 12th week. $^{*}p < 0.05$ versus control group. $^{+}p < 0.05$ versus High Fat diet group.

**Supplementary Table 3: H&E stain and IHC stain: Degree of fat and inflammation according to treatment groups**

| Groups | Degree of fat (%) | *p* value | Inflammation (Score) | *p* value | Caspase-3 (Score) | *p* value |
|---|---|---|---|---|---|---|
| C | 0 | < 0.001 | 0 | 0.116 | 0.1 ± 0.33 | < 0.001 |
| N | $74.3 \pm 25.1^{*}$ | < 0.001 | 0.8 ± 0.7 | 0.116 | 45 ± 20.7 | < 0.001 |
| G1 | 71.5 ± 24.9 | 0.999 | 1.1 ± 0.4 | 0.946 | 3.0 ± 6.4 | 0.538 |
| G2 | 77.0 ± 39.4 | 0.999 | 0.7 ± 0.7 | 0.308 | 1.2 ± 2.1 | 0.329 |
| G3 | $45.5 \pm 31.6^{+}$ | 0.049 | 1.2 ± 0.8 | 0.835 | 4.1 ± 5.6 | 0.538 |

C, control; N, high fat induced fatty liver disease; G1, G-CSF treatment once weekly from 8th to 12th week; G2, G-CSF treatment daily for 5 consecutive days in 9th week; G3, G-CSF treatment twice weekly from 9th to 12th week. $^{*}p < 0.05$ versus control group. $^{+}p < 0.05$ versus High Fat diet group.

**Supplementary Table 4: Body weight and liver weight, liver weight/body weight (LW/BW) according to treatment groups of MCD diet**

| Groups | 0 week (g) | 12 th week (g) | *p* value | Liver weight (g) | *p* value | LW /BW (%) | *p* value |
|---|---|---|---|---|---|---|---|
| C | 20.6 ± 0.5 | 33.1 ± 3.0$^{+}$ | < 0.001 | 1.55 ± 0.1 | < 0.001 | 4.7 ± 0.4 | 0.02 |
| M | 20.7 ± 0.7 | 16.0 ± 0.7$^{*}$ | < 0.001 | 0.82 ± 0.06$^{*}$ | < 0.001 | 5.2 ± 0.3 | 0.02 |
| M+G | 20.7 ± 0.5 | 16.1 ± 1.0$^{*}$ | 1 | 0.82 ± 0.09$^{*}$ | 0.952 | 5.0 ± 0.4 | 0.831 |
| M+GL | 20.9 ± 0.6 | 15.8 ± 1.2$^{*}$ | 0.999 | 0.82 ± 0.06$^{*}$ | 0.952 | 5.2 ± 0.3 | 0.991 |

C, control; MCD, methionine choline deficiency diet induced fatty liver model; M+G group, short acting G-CSF 30 μg/kg twice a week from 9th to 12th week. M+GL, long acting G-CSF 30 μg/kg once only in 9th week. $^{*}p< 0.05$ versus control group. $^{+}p< 0.05$ versus MCD diet group

**Supplementary Table 5: Biochemical parameters according to treatment groups of MCD diet**

| Groups | ALT (U/L) | AST (U/L) | T-cholesterol (mg/dl) | Glucose (mg/dl) |
|---|---|---|---|---|
| C | 41.5 ± 28.5$^{+}$ | 97.1 ± 50.2$^{+}$ | 13.7 ± 1.8$^{+}$ | 106.0 ± 8.2$^{+}$ |
| M | 346.3 ± 39.6$^{*}$ | 323.4 ± 125.0$^{*}$ | 25.6 ± 6.6$^{*}$ | 210.8 ± 26.8$^{*}$ |
| M+G | 232.6 ± 26.4$^{*}$ | 355.6 ± 66.0$^{*}$ | 27.3 ± 5.0$^{*}$ | 187.2 ± 46.8$^{*}$ |
| M+GL | 388.4 ± 153.7$^{*}$ | 272.4 ± 123.0$^{*}$ | 25.3 ± 8.8$^{*}$ | 200.5 ± 20.8$^{*}$ |

C, control; MCD, methionine choline deficiency diet induced fatty liver model; M+G group, short acting G-CSF 30 μg/kg twice a week from 9th to 12th week. M+GL, long acting G-CSF 30 μg/kg once only in 9th week. $^{*}p < 0.05$ versus control group. $^{+}p < 0.05$ versus MCD diet group.

**Supplementary Table 6: Polymerase chain reaction primer use to G-CSF experiments**

| Mouse Primer name | 5′ base 3′ |
|---|---|
| GAPDH | Forward: AGTCTACTGGTGTCTTCACC<br>Reverse: GTTGTCATATTTCTCGTGGT |
| SREBP1c | Forward: CAGAAACTCAAGCAGGAGAA<br>Reverse: GATCCTTCAGAGATTTGCTT |
| FAS | Forward: GAGTTTACTGACTCAAGGGA<br>Reverse: ACCTCAAGGTTTTATTGCCT |
| SCD-1 | Forward: AGCCAGGATTAAGAGAACTG<br>Reverse: CAGAAAACGTTTGAAACAGG |
| SREBP2 | Forward: TTTAATCAGGCTTTCTCTGG<br>Reverse: ATAAAAAGGGGTGCGTCTAT |
| HMG-CoA reductase | Forward: TCTCAATGCACTGTCTAGTG<br>Reverse: AGTCTCTGTGCAAAGAACCT |
| TNF-a | Forward: ACCCCTTTACTCTGACCCCT<br>Reverse: TGAGCCATAATCCCCTTTCT |
| MCP-1 | Forward GAAGCTGTAGTTTTTGTCAC<br>Reverse: TTTAATGTATGTCTGGACCC |
| G-CSFr | Reverse: AGGTGTATGTCCTGCCCTTG<br>Reverse: CTGGAAGGCAGAAGTGAAGG |
| GM-CSFr | Forward: CACATGCCATGAACATCACC<br>Reverse: TGAGTTCCCGGAAGCAGTAG |
| IL-1β | Forward: TCATTGTGGCTGTGGAGAAG<br>Reverse: TGTCCTCATCCTGGAAGGTC |
| IL-18 | Forward: CTGATATTGATCAAAGTGCC<br>Reverse: ACTATCCTTCACAGAGAGGG |

| Human Primer name | 5′ base 3′ |
|---|---|
| GAPDH | Forward: GTGAGGGTCTCTCTCTTCCT<br>Reverse: ACTTTGTCAAGCTCATTTCC |
| G-CSFr | Forward: GACCTGGAGGATGGAACAGA<br>Reverse: AAGGGCCTGATGTTCTCCTT |
| Gm-CSFr | Forward: AGCCCAGAGCAAAACACAGT<br>Reverse: ACTCCAGGAGCTCCAATTCA |

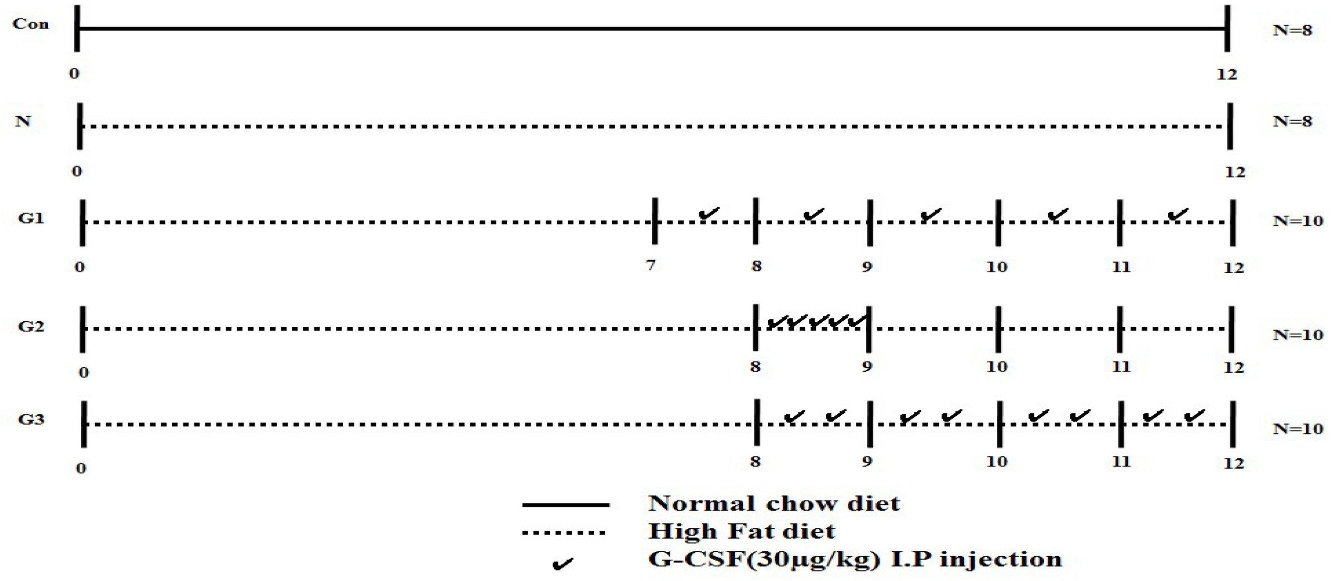


**Supplementary Figure 1: Conventional G-CSF treatment study design.**

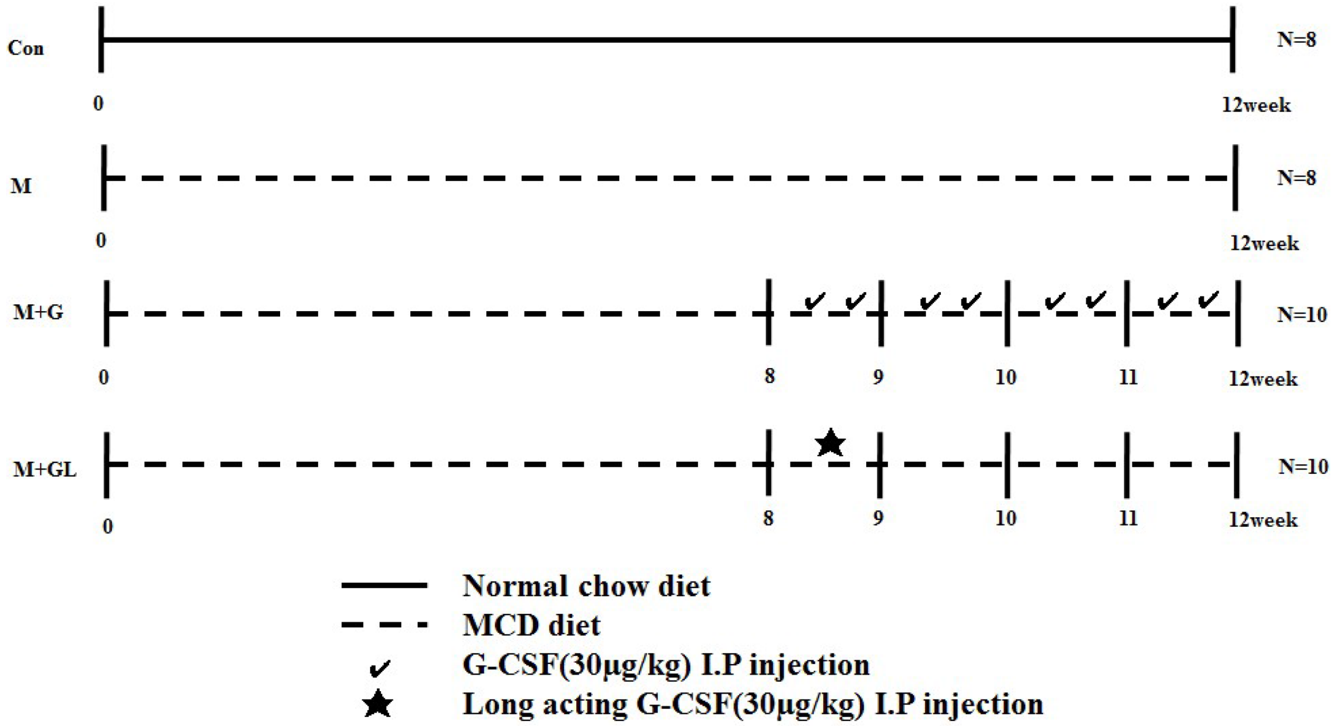


**Supplementary Figure 2: Optimization of G-CSF treatment study design.**
